# Text recycling: defining the boundaries of acceptable reuse of one’s own work

**DOI:** 10.1093/conphys/coag047

**Published:** 2026-07-10

**Authors:** Bridget O’Boyle, Essie Rodgers, Katharina Ruthsatz, Andrea Fuller

**Affiliations:** The Society for Experimental Biology, Lancaster University, Bailrigg LA1 4YW, UK; School of Environmental and Conservation Sciences, Murdoch University, 90 South Street, Murdoch, WA 6150, Australia; Ecology, Evolution, and Development Group, Department of Wetland Ecology, Doñana Biological Station, Seville, Spain; Brain Function Research Group, Department of Physiology, Faculty of Health Sciences, University of the Witwatersrand, Parktown, Johannesburg 2193, South Africa

## Introduction

Text recycling, or the re-use of one’s own previously published text, is a widespread yet underrecognized issue in academic publishing. Many authors remain uncertain about what constitutes acceptable reuse of previously published material, and why reproducing such text without appropriate acknowledgment can be problematic. Empirical studies based on large-scale computational text analyses indicate that text recycling is common across academic disciplines, particularly in STEM fields (Horbach and Halffman, 2019; Anson and Moskovitz, 2021). Plagiarism-detection software used by our journal frequently identifies text recycling in submitted manuscripts. Although some overlap may be legitimate, excessive reuse of text may result in manuscripts being returned to authors.

While the Committee on Publication Ethics (COPE) provides guidance on text recycling (Committee on Publication Ethics, 2016), awareness of these recommendations appears to be poor. In this editorial, we therefore define text recycling, explain why limits on reuse matter for the integrity of the scholarly record and offer practical guidance for authors.

## What is text recycling?

Text recycling occurs when authors re-use their own previously published work without attribution. A more formal and comprehensive definition (Text Recycling Research Project, 2020) is as follows:

“The reuse of textual materials (prose, visuals or equations) in a new document where:

- The material in the new document is identical to that of the source (or substantively equivalent in both form and content)
- The material is not presented in the new document as a quotation (via quotation marks or block indentation), and
- At least one author of the new document is also an author of the prior document.”

Readers may also come across the term ‘self-plagiarism’, and differences in opinion exist as to the exact definitions and interrelatedness of text recycling and self-plagiarism (Moskovitz, 2021; Harriman and Patel, 2014). For the purposes of this editorial, we use the term text recycling, referring to any case that matches the definition above. Text recycling is distinct from plagiarism of others’ work and from duplicate publication (i.e. publishing largely identical articles in more than one journal). These are both forms of serious academic misconduct, which are outside the scope of this editorial.

## Why editors care about text recycling?

Editors are concerned about the originality, transparency and integrity of work described in the manuscripts they receive. In the case of our journal, publications provide an evidence base that may inform conservation or management decisions and policy; redundant or misleading publications can distort syntheses, meta-analyses and applied recommendations, thereby contributing to research waste or inappropriate evidence-based decisions (Ioannidis, 2016; Puljak and Lund, 2023).

Editors also have a responsibility to uphold the journal’s reputation and ensure that published papers make a clear and meaningful contribution to the field. Distinguishing between acceptable and problematic text recycling is often not straightforward, and editorial decisions must balance considerations of transparency, originality, disciplinary norms and legitimate reuse (Hwang, 2017). Extensive re-use of text may be perceived as indicating only an incremental contribution, particularly when the text reuse is not made transparent or when it overlaps substantially with previously published work.

**Table 1:** General guidance on acceptable and unacceptable re-use of one’s own previously published text during manuscript preparation

**Type of text recycling**	**Acceptable**	**Considerations**
Copying of previously published methods, including field sampling protocol, laboratory procedures and statistical analysis approaches	Yes	Large sections of previously published methods should not be copied; instead cite the previous work, which should be checked for accuracy, and any differences noted
Study site descriptions in long-term monitoring projects	Yes	There are often no changes in the description of a researcher’s study site
Animal welfare, permitting and ethical approval statements	Yes	Precise wording is often required
Conflict of interest and data availability statements	Yes	Precise wording is often required
A limited amount of re-used text in the introduction	Yes	Large amounts of recycled text should be avoided
Previously published text in the results	No	The results should be unique to the study
A limited amount of re-used text in the discussion	Yes	The discussion should contextualize the results of the current study; large sections of overlap should not be present
Previously published text in the conclusion	No	The conclusion should be unique to the particular study
Journal articles derived from theses or technical reports	Yes	The thesis should be included in the reference list and cited in the manuscript, or included in the acknowledgements
Re-use of materials previously presented at conferences or published in conference proceedings	Yes	A reference to a conference proceeding may be included either in the acknowledgments section or in the reference list
Submission of a manuscript to a journal when the manuscript has been published on a pre-print server	Yes	Update the preprint, noting that the manuscript is under review at Journal X; add the DOI once the article is published

## When is text recycling acceptable?

Cases of text recycling should be assessed individually by journal editors (Committee on Publication Ethics, 2016, 2024; Moskovitz, 2021). Acceptability depends on several factors, including where the reuse occurs within the article, the extent of the recycled text, the article type and whether the original source has been appropriately cited (Committee on Publication Ethics, 2016, 2024; Harriman and Patel, 2014; Moskovitz, 2021). Legal considerations must also be taken into account; in some cases, text recycling may result in a breach of copyright (Committee on Publication Ethics, 2016, 2024; Text Recycling Research Project, 2020; Moskovitz, 2021).

In some contexts, limited text recycling may be appropriate and even beneficial, e.g. when describing standard experimental techniques and statistical analyses, commonly used instrumentation, or required ethical and regulatory statements (Hall *et al.*, 2021). A key consideration is whether previously published material is presented as novel, thereby potentially misleading readers about the originality of the work (Committee on Publication Ethics, 2016).

Concerns arise more often when text recycling extends to substantial reuse of material in the introduction and discussion sections, or in the conceptual framing, and without proper citation. Text recycling is not usually acceptable in results or conclusion sections of articles (Committee on Publication Ethics, 2016), where the text should reflect the specific findings and interpretation of the work in the submitted manuscript.

One issue commonly seen at *Conservation Physiology* is when authors re-use substantial sections of previously published text in the materials and methods section. As noted above (see also Table 1), text recycling may be acceptable in instances where precise or standardized wording is required. However, large, continuous sections of previously published methodological text should not be re-used. Instead, authors should cite the previous work, having first checked it for accuracy, and note any discrepancies compared to the previous publication.

To assist readers, general guidance on acceptable and unacceptable text reuse as provided by COPE (Committee on Publication Ethics, 2016, see also Hall *et al.*, 2021), is summarized in Table 1. While the guidance in Table 1 is provided as a general resource, authors should always check the guidelines of individual journals before submitting their work.

## Actions that journals take if text recycling is detected

Journal responses to text recycling will likely vary depending on editorial policy, the extent and location of the overlap, whether the original source is cited and the stage at which the issue is detected. When text recycling is identified at submission or during the peer review process, authors will usually be asked to revise the manuscript by rewriting affected sections of text, adding appropriate citations and clarifying what is novel in the submitted work. If there is extensive text recycling in multiple sections of an article, or concerns are raised about redundant publication or misleading presentation of novelty, the manuscript may be rejected without an opportunity to resubmit. If issues are detected after publication, the journal may issue a correction or an expression of concern, or in serious cases, retract the article. Legal advice may be sought if there is copyright infringement.

## Practical guidance for authors

Authors can reduce the risk of raising editorial concern about recycling of text by:

- Citing their own relevant prior publications whenever text or reasoning are reused.
- Not writing over old versions of manuscripts or pasting large sections of text directly from those manuscripts, or from theses or other reports.
- Using the cover letter to disclose overlap with related manuscripts, preprints, theses or reports during submission. The cover letter may also be used to state what is new in the current manuscript and how the work differs from earlier, closely related work, when relevant.
- Limiting verbatim reuse to sections where technical precision is needed and ensuring methodological details are tailored to the present work.
- Checking journal guidelines, and emailing the editor or editorial office if unsure of the correct approach.

## Conclusion

Text recycling is a common but overlooked issue in academic writing and requires careful consideration by authors, reviewers and editors alike. While limited and transparent reuse of text can be appropriate in specific contexts, particularly where technical precision or standardized wording is required, excessive, undisclosed or poorly contextualized reuse can undermine integrity, originality and trust in published literature. Authors should therefore treat text recycling not simply as a matter of wording, but as an issue of accurate representation: readers, reviewers and editors must be able to distinguish clearly between previously published material and the novel contributions of the submitted work. By careful adherence to journal guidelines, citing reused material where appropriate, and following the practical guidance above, authors can help ensure that their manuscripts communicate original contributions clearly, transparently and responsibly.
